# Rensching cats and dogs: feeding ecology and fecundity trends explain variation in the allometry of sexual size dimorphism

**DOI:** 10.1098/rsos.170453

**Published:** 2017-06-28

**Authors:** P. J. Johnson, M. J. Noonan, A. C. Kitchener, L. A. Harrington, C. Newman, D. W. Macdonald

**Affiliations:** 1 Wildlife Conservation Research Unit, Zoology Department, The Recanati-Kaplan Centre, University of Oxford, Tubney House, Abingdon Road, Tubney, Abingdon OX13 5QL, UK; 2 Smithsonian Conservation Biology Institute, National Zoological Park, 1500 Remount Road, Front Royal, VA 22630, USA; 3 Department of Natural Sciences, National Museums Scotland, Chambers Street, Edinburgh EH1 1JF, UK; 4 Institute of Geography, School of Geosciences, University of Edinburgh, Drummond Street, Edinburgh EH9 3PX, UK

**Keywords:** dimorphism, Felidae, Canidae, allometry, resource dispersion, diet

## Abstract

The tendency for sexual size dimorphism (SSD) to increase with body mass in taxa where males are larger, and to decrease when females are larger, is known as Rensch's rule. In mammals, where the trend occurs, it is believed to be the result of a competitive advantage for larger males, while female mass is constrained by the energetics of reproduction. Here, we examine the allometry of SSD within the Felidae and Canidae, demonstrating distinctly different patterns: in felids, there is positive allometric scaling, while there is no trend in canids. We hypothesize that feeding ecology, via its effect on female spacing patterns, is responsible for the difference; larger male mass may be advantageous only where females are dispersed such that males can defend access to them. This is supported by the observation that felids are predominately solitary, and all are obligate carnivores. Similarly, carnivorous canids are more sexually dimorphic than insectivores and omnivores, but carnivory does not contribute to a Rensch effect as dietary variation occurs across the mass spectrum. The observed inter-familial differences are also consistent with reduced constraints on female mass in the canids, where litter size increases with body mass, versus no observable allometry in the felids.

## 1. Background

Dimorphism in secondary sexual characteristics—those not directly involved with reproduction—has long attracted the attention of biologists (e.g. [1–3]). Darwin [4] was among the first to speculate on its causes, coining the term ‘sexual selection’ to describe the general tendency of males to compete for females, and its phenotypic consequences. He remarked that: ‘The law of battle for the possession of the female appears to prevail throughout the whole great class of mammals’. Intersexual difference in size (sexual size dimorphism, henceforth SSD) is a conspicuous and widespread form of dimorphism. Male-biased mass dimorphism is predominant in mammals, where sexual selection has come to be regarded as the most likely explanation for its origin [5].

Most mammals have a predominantly polygynous mating system, where sexual selection is thought to drive the observed association with SSD. In primates, monogamous species are consistently less dimorphic than polygynous species [6]. Similarly, in ruminants, species with harem-based mating systems are more dimorphic than those with territorial, polygynous and monogamous mating systems [7]. Soulsbury *et al*. [8] observed that SSD was higher in mammals with greater variation in the reproductive success of males, a measure of the degree of sexual selection. Lukas & Clutton-Brock [9] showed that mating system and SSD were linked, with male-biased SSD being more common in species where females are solitary and with their ranges overlapping compared with socially monogamous species. Reduced SSD in domestic sheep and goats compared with their wild ancestors has been attributed at least partly to the reduced importance of male combat [10].

The maintenance of male-biased SSD by sexual selection has been implicated in explaining an allometric pattern known as ‘Rensch's rule’. This rule states that, within a lineage, SSD is positively correlated with mean body mass (hyperallometry) in taxa where males are larger, and negatively correlated (hypoallometry) where females are larger (Rensch 1950 cited by Abouheif & Fairbairn [11]). Rensch's rule holds for a variety of taxa (e.g. [11–15]), but is by no means universal, and the conditions required for its expression in mammal lineages are not completely understood. For instance, while the Rensch effect holds for Mammalia as a whole, when individual groups are considered only primates [16], bovids (antelopes), cervids (deer) and macropodids (kangaroos) have shown significant Rensch allometry [15]. Conversely, for carnivorans as a whole, no Rensch effect has been observed [11,13], but this may have masked heterogeneity among families. Indeed, Webb & Freckleton [17] commented on the sensitivity of Rensch's rule to the taxonomic level investigated. Explorations of individual carnivoran families, however, are few.

In this study, we explore patterns of SSD in canids and felids, two families of Carnivora with species spanning a wide mass spectrum and differing in their dietary and socio-ecological patterns, and therefore promising for further exploration of influences on SSD. Sexual selection, although important [5], does not provide a complete explanation for patterns of SSD—it is also necessary to explain what controls female mass [14]. Indeed, the presence of the Rensch effect among breeds of domestic dog, *Canis familiaris*, shows that sexual selection cannot be the sole cause. Domestic dogs have been artificially bred for smaller mass from a common ancestor close to the wolf (*Canis lupus*) that was markedly dimorphic. A Rensch effect has been hypothesized to arise because female mass is constrained by a minimum neonate mass, so that males get smaller faster than females under selection for small mass, such that the smaller breeds are least dimorphic [18]. Where larger male mass is selected in natural populations, correlated selection for larger females will also occur due to genetic linkage [3,12,19]. But if fecundity decreases with increasing mass among female mammal species [14], a counter pressure for small female mass may reinforce a positive Rensch effect within taxa. While litter size does tend to decline with body mass in mammals as a whole [20], variation among mammalian taxa may account for some absences of a Rensch effect. In this respect, there is also a reproductive advantage for greater female mass (e.g. [21,22]). In wild canids, for example, larger species have larger litter sizes [23]—which also leads to the expectation of no Rensch effect, as has been observed in domestic canids [18].

Resource ecology is also likely to be a complicating factor: Ralls [24] speculated that the quality and dispersion of food resources could oppose polygyny by influencing the dispersion of females and therefore how individuals organize their intra- and inter-sexual territories. Any configuration of resources preventing males from defending access to females might therefore be expected to militate against a tendency for sexual dimorphism, and hence the Rensch effect. The analysis of Lukas & Clutton-Brock [9] provided some support for this; transition from the mammalian ancestral (polygynous) state to social monogamy was associated with a lower population density of individuals (adjusting for body mass); they speculated this occurred with increased competition among females, and lower female population densities. In these circumstances, it might not be possible for a male to defend more than one female, and the benefits of larger male mass would therefore tend to be lost [9]. Recent observations on mustelids and their close relatives, where a negative Rensch allometry was observed [25], support the idea that resource ecology is influential in determining whether or not a Rensch effect is observed. The explanation offered by those authors was that the smaller species tend to be wholly carnivorous, a diet hypothesized to result in a spatial configuration of females that enables males to defend access to them [26,27], which did not occur for diets with different dispersal characteristics (i.e. herbivory, insectivory and omnivory [25]).

Here, we use phylogenetically controlled analyses to explore patterns in diet, socio-ecology and fecundity relating to SSD in wild canids and felids. Felids are all obligate carnivores, and while it may be an over-simplification to allude to a ‘typical felid social system’ [28], most are solitary. Canids, by contrast, vary considerably in feeding ecology, but monogamy is dominant. Previous studies have found no evidence for a Rensch effect in canids [29] and an inconclusive pattern in felids [30]. The study of Bideau & Martinez [29] did not investigate the effect of diet. We explore how body mass and diet type are related to sexual dimorphism *per se* and to the Rensch effect in these families. We also explore how that part of fecundity indexed by litter size changes with body mass in these groups, as the overall positive Rensch effect in mammals has been said to be based in part on reproductive constraint on female mass.

## 2. Material and methods

The data on sexual dimorphism were obtained from the monographs on wild canids and felids by Macdonald & Loveridge [31] and Macdonald & Sillero-Zubiri [32], respectively, and augmented as detailed in the electronic supplementary material. Data concerning diet and social system were taken from the life-history dataset used by Noonan *et al*. [33]. Diet and social system were treated as categorical predictors in statistical models. Diet with levels: ‘carnivorous’, ‘omnivorous’ and ‘insectivorous’, and social class with levels: ‘1’, ‘Solitary’; ‘2’, ‘Pairs’; ‘3’, ‘Groups’; ‘4’, ‘Social’. Because, social classes ‘1’ and ‘3’ were scarce, these were aggregated with ‘2’ and ‘4’, respectively. Social class and diet data were used only for canids, the felids being dominated by solitary systems and carnivorous diets.

For consistency with previous studies, we quantified SSD as the ratio of mean male to mean female masses [11]. Following [12,13], we regressed log_e_ male mass (the dependent variable in our models) against log_e_ female mass. We tested our null hypothesis of no allometric trend using the slope of this regression; slopes of greater than 1.0 are consistent with conventional positive Rensch allometry, and where slope confidence intervals do not include zero, the Ho of no effect is rejected at the 0.05 level. We used a model 1 regression; model 1 and model 2 solutions converge as the correlation between male mass and female mass approaches 1.0 [17]. The correlation was 0.994 for felids and 0.996 for canids. For canids, we treated diet and social class as categorical predictors in our models, with SSD used as the response. We also tested if body mass was related to diet for canids, aggregating non-carnivores in the same class to avoid sparse classes, and with female mass as the response.

We used the R (CRAN) ‘*MCMCglmm*’ package [34] to control for phylogenetic dependencies. Phylogenetic relationships for the Felidae were taken from a recent consensus phylogeny of the Carnivora [35]. For the Canidae, however, we took relationships from Lindblad-Toh *et al*. [36] because its tree was better resolved and included more species. The programme derives parameter estimates using a Bayesian framework; uninformative, inverse gamma priors were applied, as in [33]. The number of model iterations, thinning interval and burn-in period were determined using diagnostic tests in the *R* package ‘*coda*’ [37], and convergence was confirmed using the Geweke diagnostic [38].

## 3. Results

**Figure 1.**
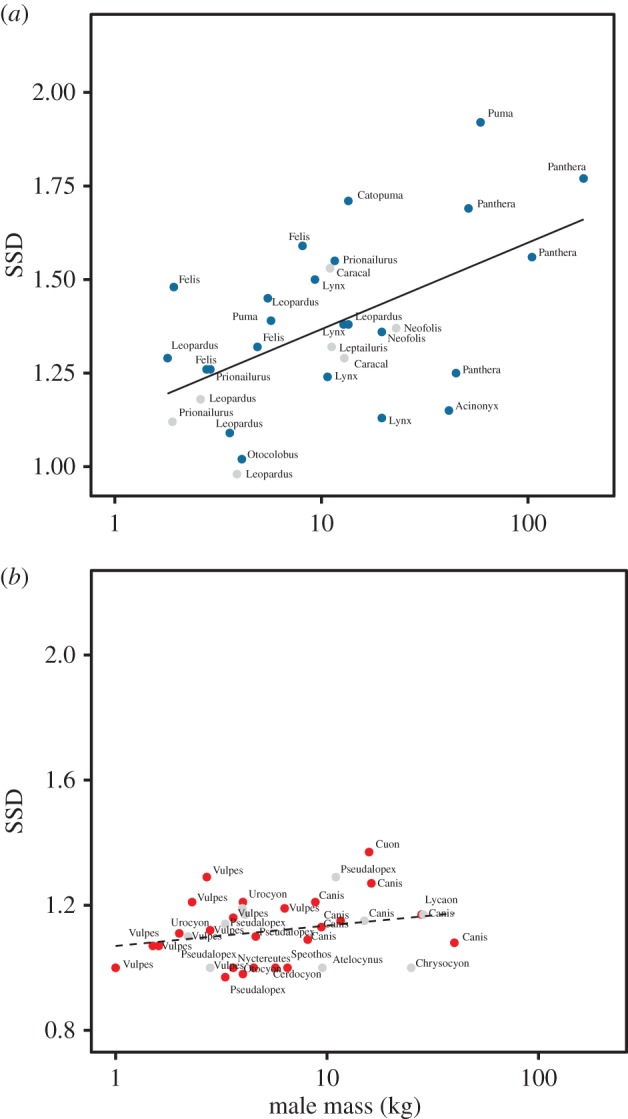
Sexual dimorphism and female mass in (*a*) felids and (*b*) canids. (Species plotted as grey points do not appear in the phylogeny.)

In felids, the MCMCglmm slope of male mass versus female mass (log scale) was 1.07 (CI 1.01–1.12), indicating a slope significantly greater than 1.0 and a conventional positive Rensch trend (figure 1*a*). In canids, the slope was close to 1.0, and there was no evidence for a Rensch effect (figure 1*b*, 0.99, CI 0.95–1.06).

**Figure 2.**
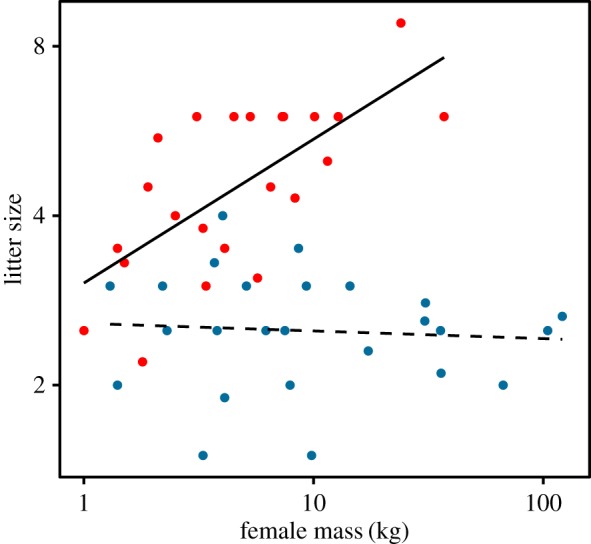
Litter size and female mass in felids (blue points) and canids.

Litter size scaled positively with body mass in canids (figure 2, slope = 0.29, CI 0.13–0.45, pMCMC = 0.002). The slope was lower for carnivorous canids alone (0.10, CI −0.06 to 0.30). There was no trend for felids (figure 2, slope = 0.02, CI −0.09 to 0.13, pMCMC = 0.68).

**Figure 3.**
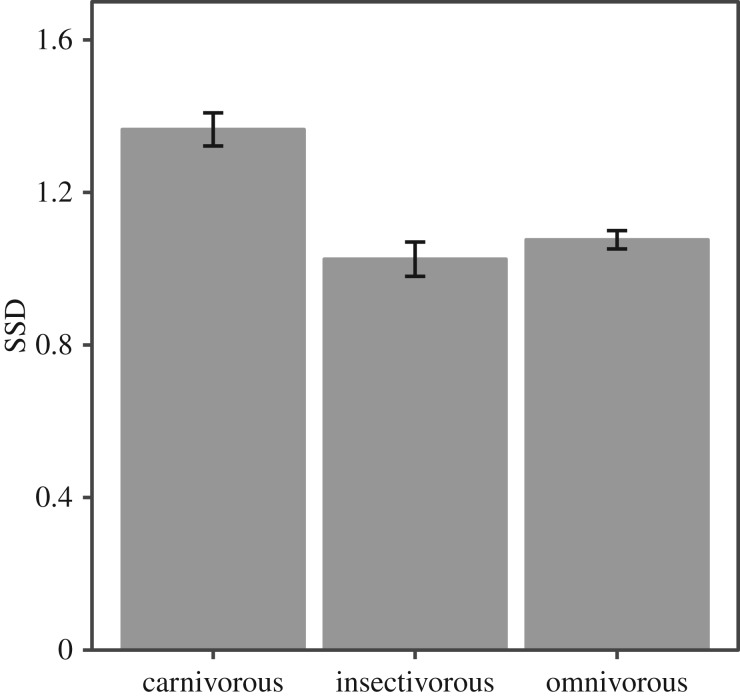
Sexual dimorphism and dietary class in canids (means with CI).

Carnivorous canid species were more sexually dimorphic than other dietary categories combined (figure 3, difference = 0.07, CI −0.009 to 0.14, pMCMC = 0.07) and were also larger (difference = 6.47, CI 0.5 – 12.5, pMCMC = 0.03). There was no evidence for a Rensch effect in this subgroup (slope = 0.95, CI 0.87–1.04, *N* = 10 species), or any evidence that canid social system was related to sexual dimorphism (difference = −0.03, CI −0.16 to 0.08, pMCMC = 0.60).

## 4. Discussion

Here, we demonstrate a positive Rensch effect among wild felids, which has not previously been shown, and confirm the absence of the effect in canids. We also demonstrate a link between SSD and diet—the most carnivorous canids were more dimorphic, and were also larger. The effect of size and diet on SSD is therefore difficult to disentangle. We note that a previous study [30], reporting no Rensch effect in felids after phylogenetic correction, used different biomass values and also included a number of subspecies, which may have influenced the authors' conclusions. Increasing litter size with body mass in canids, bearing in mind that this is not a complete measure of fecundity [20], suggests no constraint attributable to this part of fecundity for this group; indeed, it suggests the opposite effect, that larger mass is favoured. Carnivorous canid species also followed this overall pattern, which clearly militates against the expression of a Rensch effect in this group.

While felids and canids are not very closely related, and we cannot exclude the possibility that confounding variables are correlated with the observed effects, these observations support the idea that the presence of a Rensch effect in carnivorans may arise at least in part as a result of diet affecting sexual selection. We observe here that the trend in musteloids for SSD to be associated with carnivory [25] is mirrored among canids: the most sexually dimorphic canids are also the most carnivorous (figure 3). This does not scale with body mass (figure 1) because the species with SSD below 1.1 span the range of body masses in the group. These are almost all omnivores and insectivores; only one (with SSD < 1.1), the bush dog (*Speothos venaticus*), is principally carnivorous. The difference between felids and canids may also result from the tendency for canids to exploit large prey by forming groups rather than by evolving larger individual body mass, which is the felid pattern; the scaling of body mass with prey mass is much looser in canids [28]. Group-hunting species at the upper end of the mass spectrum, conspicuously African wild dogs (*Lycaon pictus*) and grey wolves (*C. lupus*), have relatively low SSD (figure 2). This tendency was also observed in musteloids, where, the social otters, for example, have low SSD [25].

For a link between diet and sexual dimorphism to be part of a general explanation for the expression of Rensch's rule in Carnivora, it would be necessary to explain how a carnivorous diet frequently allows males to compete for access to females, and therefore, for there to be an advantage of larger body mass. The evidence for this is as yet indirect and incomplete. For felids, accounts of the social systems of species are often fragmentary and awaiting elucidation by molecular work [28]. While patterns of overlap of both male and female home ranges vary, male ranges are generally substantially larger than those of females (table 5.2 in [28]). This is comparable to the mammalian ancestral system where females are sedentary and males are ‘roaming’ [9]. Under this system, we would expect male mass to be selected for. Lukas & Clutton-Brock [9] provide some evidence that shifts from this system to social monogamy are associated with resource effects; the ancestral system may tend to give way to social monogamy and lower sexual dimorphism when the diet is of lower nutritional quality. Our observation that carnivorous canids are more dimorphic than omnivores and insectivores is consistent with this, though we did not find any evidence that social system was consistently related to sexual dimorphism. Resource effects may be more complicated; patchiness and predictability may form an important part of the explanation for patterns in sexual dimorphism but are not easily measured in nature.

## 5. Conclusion

While the Felidae follow a positive Rensch allometry, exhibiting greater SSD among larger species, the Canidae show no significant trend. We attribute these differences to variation in feeding ecology, where the dispersion of resources for omnivorous and insectivorous species precludes mating systems where defending access to multiple females is a viable male strategy [25,27]. This is supported by the observation that felids are predominately solitary, and all are obligate carnivores. Similarly, carnivorous canids are more sexually dimorphic than insectivores and omnivores, but carnivory does not contribute to a Rensch effect as dietary variation occurs across the mass spectrum. The observed inter-familial differences are also consistent with reduced constraints on female mass in the canids, where litter size increases with body mass, versus no observable allometry in the felids. We conclude that diet and resource dispersion can promote social and mating systems that undermine the advantage of large male size, by reducing the extent to which contest competition contributes to male reproductive success.

## Supplementary Material

Data used in ‘results’ section

## Data accessibility

The data used in these analyses are provided as the electronic supplementary material.

## References

[1] Fairbairn DJ, Blanckenhorn WU, Székely T. 2007 Sex, size, and gender roles: evolutionary studies of sexual size dimorphism, ix, 266 p Oxford, UK: Oxford University Press.

[2] Hedrick AV, Temeles EJ 1989 The evolution of sexual dimorphism in animals—hypotheses and tests. Trends Ecol. Evol. 4, 136–138. (doi:10.1016/0169-5347(89)90212-7) 21227335 10.1016/0169-5347(89)90212-7

[3] Lande R 1980 Sexual dimorphism, sexual selection, and adaptation in polygenic characters. Evolution 34, 292–305. (doi:10.1111/j.1558-5646.1980.tb04817.x) 28563426 10.1111/j.1558-5646.1980.tb04817.x

[4] Darwin C 1871 The descent of man, and selection in relation to sex. London, UK: John Murray.

[5] Isaac JL 2005 Potential causes and life-history consequences of sexual size dimorphism in mammals. Mammal Rev. 35, 101–115. (doi:10.1111/j.1365-2907.2005.00045.x)

[6] Clutton-Brock TH, Harvey PH, Rudder B 1977 Sexual dimorphism, socionomic sex ratio and body weight in primates. Nature 269, 797–800. (doi:10.1038/269797a0) 927503 10.1038/269797a0

[7] Weckerly FW 1998 Sexual-size dimorphism: influence of mass and mating systems in the most dimorphic mammals. J. Mammal. 79, 33–52. (doi:10.2307/1382840)

[8] Soulsbury CD, Kervinen M, Lebigre C 2014 Sexual size dimorphism and the strength of sexual selection in mammals and birds. Evol. Ecol. Res. 16, 63–76.

[9] Lukas D, Clutton-Brock TH 2013 The evolution of social monogamy in mammals. Science 341, 526–530. (doi:10.1126/science.1238677) 23896459 10.1126/science.1238677

[10] Polak J, Frynta D 2009 Sexual size dimorphism in domestic goats, sheep, and their wild relatives. Biol. J. Linn. Soc. 98, 872–883. (doi:10.1111/j.1095-8312.2009.01294.x)

[11] Abouheif E, Fairbairn DJ 1997 A comparative analysis of allometry for sexual size dimorphism: assessing Rensch's rule. Am. Nat. 149, 540–562. (doi:10.1086/286004)

[12] Dale J, Dunn PO, Figuerola J, Lislevand T, Szekely T, Whittingham LA 2007 Sexual selection explains Rensch's rule of allometry for sexual size dimorphism. Proc. R. Soc. B 274, 2971–2979. (doi:10.1098/rspb.2007.1043) 10.1098/rspb.2007.1043 PMC2211517 17878139

[13] Fairbairn DJ 1997 Allometry for sexual size dimorphism: pattern and process in the coevolution of body size in males and females. Annu. Rev. Ecol. Syst. 28, 659–687. (doi:10.1146/annurev.ecolsys.28.1.659)

[14] Lindenfors P, Gittleman JL, Jones KE 2007 Sexual size dimorphism in mammals. In Sex, size and gender roles: evolutionary studies of sexual size dimorphism (eds Fairbairn DJ, Blanckenhorn WV, Szekely T). Oxford, UK: Oxford University Press.

[15] Sibly RM, Zuo WY, Kodric-Brown A, Brown JH 2012 Rensch's rule in large herbivorous mammals derived from metabolic scaling. Am. Nat. 179, 169–177. (doi:10.1086/663686) 22218307 10.1086/663686

[16] Lindenfors P, Tullberg BS 1998 Phylogenetic analyses of primate size evolution: the consequences of sexual selection. Biol. J. Linn. Soc. 64, 413–447. (doi:10.1111/j.1095-8312.1998.tb00342.x)

[17] Webb TJ, Freckleton RP 2007 Only half right: species with female-biased sexual size dimorphism consistently break Rensch's rule. PLoS ONE 2, e897 (doi:10.1371/journal.pone.0000897) 17878932 10.1371/journal.pone.0000897 PMC1964802

[18] Frynta D, Baudsoya J, Hradcova P, Faultsoava K, Kratochvil K 2012 Allometry of sexual size dimorphism in domestic dog. PLoS ONE 7, 1–6. (doi:10.1371/journal.pone.0046125) 10.1371/journal.pone.0046125 PMC3458007 23049956

[19] Kemper KE, Visscher PM, Goddard ME. 2012 Genetic architecture of body size in mammals. Genome Biol. 13, Artn 244 (doi:10.1186/Gb-2012-13-4-244) 10.1186/gb-2012-13-4-244 PMC3446298 22546202

[20] Allaine D, Pontier D, Gaillard JM, Lebreton JD, Trouvilliez J, Clobert J 1987 The relationship between fecundity and adult body-weight in homeotherms. Oecologia 73, 478–480. (doi:10.1007/Bf00385268) 28311533 10.1007/BF00385268

[21] Garcia-Navas V, Bonnet T, Bonat R, Postma E 2015 The role of fecundity and sexual selection in the evolution of size and sexual size dimorphism in New World and Old World voles (Rodentia: Arvicolinae). Oikos 125, 1250–1260. (doi:10.1111/oik.03026)

[22] Zhao L, Chen YJ, Lou SL, Huang Y, Jehle R, Liao WB 2016 Reciprocal sexual size dimorphism and Rensch's rule in toad-headed lizards, *Phrynocephalus vlangalii*. Salamandra 52, U261–U291.

[23] Moehlman PD, Hofer H 1997 Cooperative breeding, reproductive suppression, and body size in canids. In Cooperative breeding in mammals (eds Solomon NG, French JA). Cambridge, UK: Cambridge University Press.

[24] Ralls K 1977 Sexual dimorphism in mammals—avian models and unanswered questions. Am. Nat. 111, 917–938. (doi:10.1086/283223)

[25] Noonan MJ, Johnson PJ, Kitchener AC, Harrington LA, Newman C 2016 Sexual size dimorphism in musteloids: an anomalous allometric pattern is explained by feeding ecology. Ecol. Evol. 6, 8495–8501. (doi:10.1002/ece3.2480) 28031801 10.1002/ece3.2480 PMC5167046

[26] Macdonald DW 1983 The ecology of carnivore social behaviour. Nature 301, 379–384. (doi:10.1038/301379a0)

[27] Macdonald DW, Johnson DDP 2015 Patchwork planet: the resource dispersion hypothesis, society, and the ecology of life. J. Zool. 295, 75–107. (doi:10.1111/jzo.12202)

[28] Macdonald DW, Mosser A 2010 Felid society. In The biology and conservation of wild felids (eds Macdonald DW, Loveridge AJ), pp. 125–160. Oxford, UK: Oxford University Press.

[29] Bideau CJ, Martinez PA 2016 Sexual size dimorphism and Rensch's rule in Canidae. Biol. J. Linn Soc. 119, 816–830. (doi:10.1111/bij.12848)

[30] Martinez PA, Ferreira Armado T, Bideau CJ 2014 Una apoxmacion filogenetica al estudio del dimorphismo sexual de tamano en felidae y la evalucion de la regla de Rensch. ecosistemas 23, 27–36. (doi:10.7818/ECOS.2014.23-1.05)

[31] Macdonald DW, Loveridge AJ 2010 The biology and conservation of wild felids, p. 762 Oxford, UK: Oxford University Press.

[32] Macdonald DW, Sillero-Zubiri C 2004 The biology and conservation of wild canids, p. 450 Oxford, UK: Oxford University Press.

[33] Noonan MJ, Newman C, Buesching CD, Macdonald DW 2015 Evolution and function of fossoriality in the carnivora: implications for group-living. Front. Ecol. Evol. 3, 116 (doi:10.3389/fevo.2015.00116)

[34] Hadfield JD 2010 MCMC methods for multi-response generalized linear mixed models: the MCMCglmm R package. J. Stat. Softw. 33, 1–22. (doi:10.18637/jss.v033.i02)20808728

[35] Agnarsson I, Kuntner M, May-Collado LJ 2010 Dogs, cats, and kin: a molecular species-level phylogeny of Carnivora. Mol. Phylogenet. Evol. 54, 726–745. (doi:10.1016/j.ympev.2009.10.033). 19900567 10.1016/j.ympev.2009.10.033

[36] Lindblad-Toh K et al. 2005 Genome sequence, comparative analysis and haplotype structure of the domestic dog. Nature 438, 803–819. (doi:10.1038/nature04338) 16341006 10.1038/nature04338

[37] Plummer M, Best N, Cowles K, Vine K 2006 Convergence diagnosis and output analysis for MCMC. R News 6, 7–11.

[38] Geweke J 1992 Evaluating the accuracy of sampling-based approaches to the calculation of posterior moments. In Bayesian statistics 4: Proceedings of the fourth Valencia international meeting (eds Bernardo M, Berger J, Dawid AP, Smith AFM). Oxford, UK: Oxford University Press.

